# Interventional treatment for littoral cell angioma of spleen-induced thrombocytopenia: a case report and literature review

**DOI:** 10.3389/fonc.2026.1820397

**Published:** 2026-05-12

**Authors:** Zihao Wu, Kunshan Chen, Zhenyin Liu

**Affiliations:** Department of Interventional Radiology and Vascular Anomalies, Guangzhou Women and Children’s Medical Center, Guangzhou Medical University, Guangzhou, Guangdong, China

**Keywords:** immunohistochemistry, littoral cell angioma, sclerotherapy, splenectomy, splenic neoplasms

## Abstract

Littoral cell angioma (LCA) is a primary vascular tumor originating from the cells lining the sinuses of the red pulp. This case presents a 1-year-old male with thrombocytopenia incidentally detected by ultrasound as an isolated LCA. The tumor was evaluated via ultrasound, computed tomography, and biochemical testing. The patient underwent interventional embolization, with pathological examination confirming LCA diagnosis. Postoperatively, the lesion showed a stable reduction in size, platelet counts normalized, and no complications or recurrence occurred. This case analyzes the clinical, imaging, and pathological features of this rare splenic tumor, aiming to explore the clinical significance of interventional embolization for LCA with thrombocytopenia.

## 1 Introduction

LCA is a rare benign vascular tumor of the spleen, originating from the cells lining the sinuses of the red pulp (1). Falk et al. (2) first described and named LCA in 1991. To date, only a few hundred cases have been reported worldwide (2, 3). LCA predominantly affects middle-aged individuals, with no gender difference (4). The patient’s symptoms and signs primarily include splenomegaly, hypersplenism, thrombocytopenia, and anemia. Due to the atypical clinical symptoms and imaging features of LCA, it is often misdiagnosed as hemangioma or lymphoma before surgery. Pathological biopsy remains the gold standard for diagnosis (5). Approximately 30% of LCA patients have concomitant malignancies, primarily involving the digestive system, such as colorectal cancer, pancreatic cancer, and hepatocellular carcinoma (6). Some LCA cases are associated with immune system disorders, including lymphocytic colitis, ankylosing spondylitis, systemic lupus erythematosus, and chronic glomerulonephritis. Some researchers think that this immune system issue may be related to the cause of LCA (7). The selection of treatment modalities for LCA currently relies primarily on splenectomy in clinical practice. This procedure not only removes the spleen but also provides ample biopsy tissue for pathological diagnosis. In this case, we employed interventional sclerotherapy. Regarding literature on interventional sclerotherapy, one case involved a patient with LCA complicated by splenomegaly. Blood tests revealed markedly reduced platelet counts. The physician opted for a relatively conservative interventional approach. Following splenic artery embolization, follow-up showed platelet counts gradually returning to normal levels. No recurrence of the lesion was observed, indicating favorable treatment outcomes (8). The following report and discussion pertain to a case of LCA diagnosed and treated by our hospital’s Interventional Hemangioma Department in July 2025.

## 2 Case report

### 2.1 Case information

A male patient, approximately 1 year old, was incidentally found to have a splenic mass measuring approximately 34 mm×35 mm during abdominal ultrasound examination as part of a routine physical examination. Physical examination revealed no palpable abdominal mass, and the abdomen showed no tenderness or rebound tenderness. The patient exhibited no significant symptoms such as abdominal pain, nausea, vomiting, or fever. March 8, 2025: CT imaging at another hospital suggested LCA. March 8, 2025: Biochemical tests at another hospital indicated decreased platelets: 156×10^9/L. March 11, 2025: Platelets further decreased: 107×10^9/L. On June 20, 2025, biochemical tests at an external hospital revealed a further decrease in platelet count: 64×10^9/L. No medication, invasive tests, or treatments were administered at the external hospital. The patient presented to our hospital’s Interventional Vascular Oncology Department on July 1, 2025, for further diagnostic clarification and treatment. Admitted with a diagnosis of “splenic tumor”. The patient is in generally fair condition, presenting with mild abdominal distension but no significant symptoms or discomfort. No signs of infection, such as fever, are present. No abdominal tenderness or rebound tenderness is noted, and no palpable abdominal mass is detected.

### 2.2 Preoperative biochemical and imaging examinations

**Figure 1:**
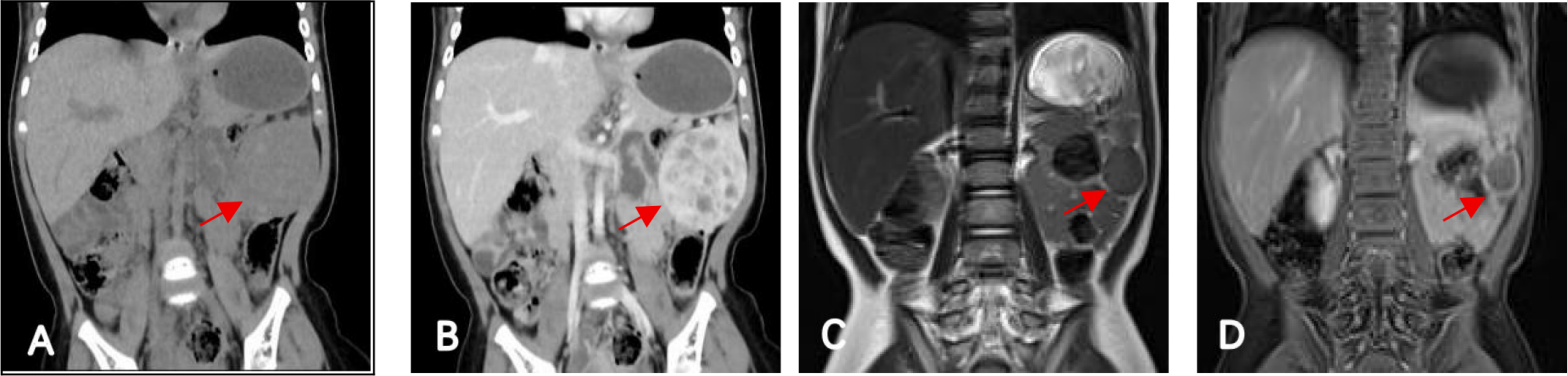
**(A)** Non-contrast CT scan reveals a round, slightly hypodense mass at the inferior pole of the spleen with well-defined borders, measuring approximately 4.5 cm×4.7 cm×4.6 cm. **(B)** The spleen mass demonstrates marked peripheral enhancement with multiple internal hypodense areas showing no enhancement. **(C, D)** At 9 months post-treatment, follow-up MRI revealed a round abnormal signal lesion within the spleen measuring approximately 2.2 cm×2.1 cm×2.2 cm, showing a significant reduction in size compared to previous findings. The lesion had well-defined margins, exhibited slightly hypointense signal intensity on T2WI, and demonstrated marked periphery enhancement on T1-weighted contrast-enhanced scanning.

Preoperative biochemical test results indicate that on July 1, 2025, the platelet count was significantly below the normal range: 88×10^9/L. Tumor markers: Squamous cell carcinoma antigen was slightly elevated: 2.17 ng/ml. All other blood biochemical and tumor marker tests were within normal ranges. On July 2, 2025, the patient underwent preoperative non-contrast and contrast-enhanced abdominal CT scanning at our institution. Imaging findings revealed a solitary, mildly hypodense, round lesion within the spleen, measuring approximately 4.5 cm× 4.7 cm× 4.6 cm, with progressive and marked peripheral enhancement (**Figure 1**).

### 2.3 Treatment strategy

**Figure 2:**
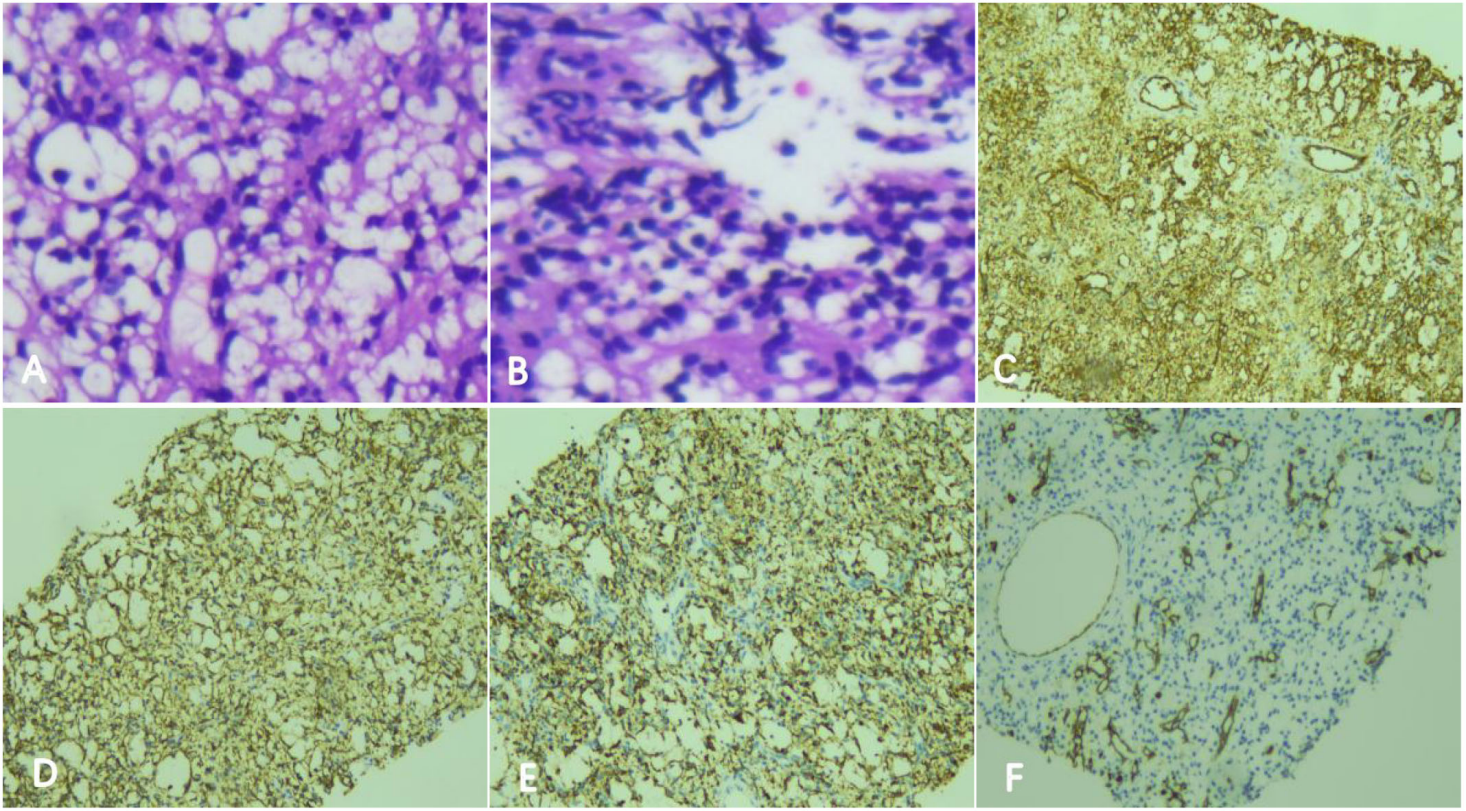
**(A, B)** Microscopic examination of pathological biopsy tissue reveals a predominance of thin-walled small vessels, with some vessels showing dilation and cuboidal endothelial cells protruding into the lumen. Fibroblasts proliferate between the vessels, accompanied by scattered inflammatory infiltration. Localized infarcts and sporadic hemosiderin deposits are observed. Immunohistochemistry revealed a splenic tumor with positive immunohistochemical profiles of CD8-, CD31+, CD163+, CD68+ and CD34+ cells. Immunohistochemical results: **(C)** endothelial marker CD31 (CD31 stain, x200); **(D)** endothelial marker CD163 (CD163 stain, x200); **(E)** endothelial marker CD68 (CD68 stain, x200); **(F)** endothelial marker CD34 (CD34 stain, x200).

**Figure 3:**
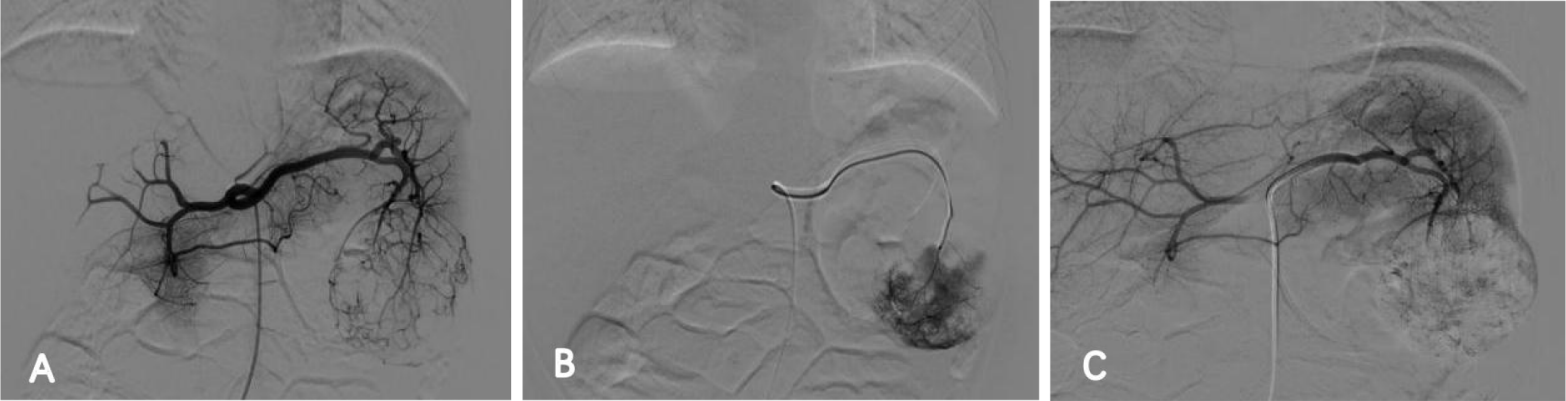
**(A)** Selective insertion of a catheter into the celiac artery for angiography under DSA guidance, demonstrating splenic mass supplied by splenic artery branches; **(B)** under DSA fluoroscopy, slow injection of a sclerosant mixture (2 ml 3% polidocanol foam agent, 8 mg pyramycin) and 350 µm PVA particles via microcatheter for sclerotherapy embolization; **(C)** final repeat DSA abdominal artery angiography shows near-complete disappearance of lesion enhancement with no contrast extravasation.

On July 3, 2025, the patient underwent interventional therapy. Following general anesthesia, selective insertion of a catheter into the celiac artery was performed under DSA guidance, revealing splenic tumor supply from splenic artery branches. After ultra-selective targeting of the tumor artery, a sclerotherapy mixture (2 ml 3% polidocanol foam agent and 8 mg pyramycin) and 350 µm PVA particles were injected via microcatheter for sclerotherapy embolization. Under ultrasound and DSA guidance, a biopsy needle was advanced through the left abdominal wall into the mass. The obtained tissue samples were used for histopathological and immunohistochemical (IHC) examinations (**Figure 2**). A final DSA angiography confirmed near-complete disappearance of contrast enhancement within the lesion with no extravasation, concluding the procedure (**Figure 3**).

## 3 Results

### 3.1 Pathological biopsy and immunohistochemical staining examination

### 3.2 Follow-up

On July 4, 2025, the patient’s platelet count remained below normal at 105×10^9/L on the first postoperative day. On the second postoperative day, the platelet count was 131×10^9/L. An ultrasound follow-up on August 22, 2025, indicated that the splenic mass had decreased in size compared to previous measurements, measuring approximately 42 mm×35 mm. No significant blood flow signals were observed within the tumor. On 2025-8-29, after a follow-up blood test showed platelets within normal range (508×10^9/L), medication was discontinued. On 2025-9-12, a follow-up blood test at another hospital indicated platelets remained within normal range at 248×10^9/L. Ultrasound follow-up on 2025-12–3 showed further reduction in the splenic mass to approximately 28 mm × 25 mm, with no significant abnormal blood flow signals detected within the tumor. Blood count follow-up on 2025-12–3 maintained platelets within the normal range at 289×10^9/L. The platelet count changes were plotted as a line graph for easier observation (**Table 1**). Postoperative non-contrast and contrast-enhanced abdominal MRI follow-up examinations performed on March 29, 2026, demonstrated further reduction in the lesion volume to approximately 2.2 cm × 2.1 cm × 2.2 cm (**Figure 1**).

**Table 1:** The progression of platelet changes.

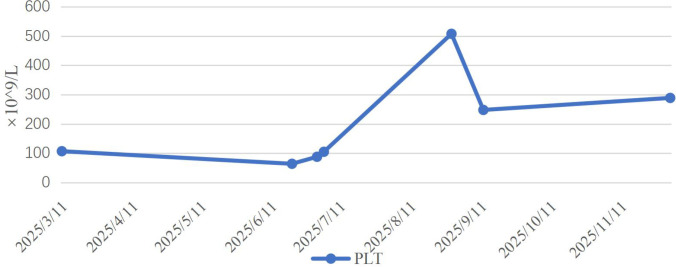

The progression of platelet changes. On June 20, 2025, the patient’s platelet count decreased to the lowest value recorded in laboratory tests: 64×10^9/L. On July 3, 2025, the patient underwent interventional therapy. On July 4, 2025, the platelet count on the first postoperative day remained below the normal range: 105×10^9/L. On August 29, 2025, follow-up blood tests showed that the platelet count was within the normal range (508×10^9/L). On September 12,2025, follow-up blood test results showed that the platelet count was within the normal range (248×10^9/L). Subsequent follow-up examinations consistently maintained the platelet count within the normal range. The most recent follow-up blood test, performed on December 3, 2025, showed that the platelet count remained within the normal range (289×10^9/L).

## 4 Discussion

LCA originates from the cells lining the sinuses of the red pulp in the spleen. It is most commonly observed in middle-aged individuals, with a median age of onset at 50 years. There is no significant difference in incidence between males and females (4, 9). LCA typically presents as a multifocal disease, and its manifestation as an isolated mass in adolescents is particularly rare (10). Patients with LCA may also have malignant tumors in other sites or immune system-related diseases, with colorectal cancer, lymphoma, and lymphocytic leukemia, and renal cell carcinoma ranking as the top three in incidence (7, 11). Among the 25 LCA patients studied by Peskova et al. (6), 15 also had tumors in other organs. The pediatric patient in this report has no other tumors or related medical history, and no tumors in other organs have been detected during the current short-term follow-up period. The specific pathogenesis of LCA remains unclear at present. Clinical manifestations of LCA are atypical and may present as abdominal masses, abdominal pain, bloating, weight loss, and other discomforts. Laboratory tests for LCA may reveal a pancytopenia in some patients (12). This pediatric patient primarily presented with a marked decrease in platelet count, with the lowest recorded value being 64×10^9^/L. LCA on CT imaging: On non-contrast scans, lesions appear as round or oval hypodense foci, which may show marked peripheral enhancement after contrast administration. Due to its rarity and non-specific imaging features, LCA is often misdiagnosed preoperatively as splenic lymphoma or splenic hemangioma. In this report, the patient was initially misdiagnosed as having a splenic hemangioma based on imaging studies. Pathological examination reveals characteristic changes: interconnected vascular spaces lined by a single layer of endothelial-like cells. Immunohistochemistry demonstrates dual differentiation, with positive expression of both endothelial and histiocytic markers (e.g., CD31, CD34 and CD68). Typically, CD8 should be negative; however, some researchers report and suggest that CD8(+) expression may indicate a higher risk of postoperative recurrence (13).

The primary treatment strategy for LCA is splenectomy, which may be performed via laparoscopic splenectomy or open splenectomy, depending on the location and size of the nodules (14). Some scholars have proposed that partial splenectomy may be performed for patients with solitary nodules to preserve splenic function and prevent the occurrence of life-threatening infections following total splenectomy (15). Regarding the widespread experience with interventional embolization for tumors originating from splenic vascular sources, while both “hemangioma” and “LCA” originate from the splenic vascular/reticuloendothelial system, they differ in pathology, origin, and mode of progression. Therefore, embolization experience with hemangiomas cannot be directly applied to LCA. For patients with LCA, only a few foreign case reports have documented the use of interventional embolization as the sole treatment modality. To date, no domestic reports have documented the use of interventional embolization as the sole treatment for LCA. Mac New et al. (16) reported a case of partial splenectomy via the inferior vena cava (IVC) approach with favorable postoperative recovery. However, given the potential malignancy of IVC tumors and the difficulty in preoperative diagnosis, partial splenectomy may offer limited long-term health benefits for patients (9). Additionally, some case reports suggest that LCA may harbor malignant potential. Ben-Izhak et al. (17) described a patient with LCA who developed multiple metastases in the abdomen and liver eight years after splenectomy, with pathological confirmation of their origin from the LCA.

Given the immunological requirements of the spleen in infants and young children, this case adopted a minimally invasive, highly reproducible, and relatively conservative interventional embolization treatment protocol. We prioritized superselective embolization to address the patient’s significantly reduced platelet count while preserving splenic function in infants, thereby avoiding severe immunodeficiency, controlling the extent of splenic infarction, preventing severe infections, and mitigating the risk of ectopic embolization. Postoperative follow-up closely monitored biochemical indicators and imaging changes. Approximately six months postoperatively, the patient’s biochemical parameters remain stable, and imaging studies indicate sustained reduction of the lesion. Long-term follow-up is required to assess the long-term efficacy of this interventional procedure. The author believes that while splenectomy remains the primary treatment for LCA, it carries risks of postoperative embolization, metastasis, recurrence, and malignant transformation. In cases where symptoms and biochemical indicators require active management or the patient cannot tolerate surgery, prioritizing interventional embolization offers a reasonable, flexible approach. This not only reduces lesion size but also serves as an adjunctive preoperative measure to shrink tumor volume, thereby facilitating subsequent surgical intervention. However, as previously noted, substantial gaps remain in clinical data regarding the efficacy of interventional embolization for LCA and long-term follow-up observations both domestically and internationally. This calls for collaborative efforts among scholars to explore more optimal solutions.

## 5 Conclusion

LCA is a rare benign splenic vascular tumor with an unclear pathogenesis. Clinically, LCA is prone to preoperative misdiagnosis, and its confirmation relies on postoperative pathological biopsy and immunohistochemical examination. Splenectomy remains the most common treatment option, but it is important to note that LCA carries a potential risk of malignant transformation and is often associated with malignancies in other organs, necessitating close postoperative follow-up. After careful consideration of the patient’s condition, we employed an uncommon therapeutic approach—interventional sclerotherapy embolization—for a splenic LCA case at our institution. Postoperatively, the lesion demonstrated stable volume reduction, and the patient’s markedly decreased platelet count was effectively controlled. This experience aims to contribute insights toward developing safer, more effective, and patient-friendly treatment strategies.

## Data Availability

The original contributions presented in the study are included in the article/supplementary material. Further inquiries can be directed to the corresponding author.
